# Supplementary material for: Understanding healing: A comparative analysis in chronic diseases with leprosy—A scoping review
Source: PLoS Negl Trop Dis. 2026 Mar 2;20(3):e0013748. doi: 10.1371/journal.pntd.0013748 (PMC12962515; doi:10.1371/journal.pntd.0013748)
Supplement: S1 Table — This table presents detailed information on all 85 studies included. The table corresponds to PRISMA-ScR checklist item 15. (DOCX) [file pntd.0013748.s001.docx]

**S1 Table : Characteristics of included studies in the scoping review.**
 Summary of studies included for Tuberculosis.

| Reference (1st author and year) | Design | Location | Participants  Study population  total (M:F) | Tools | Themes looked at |
| --- | --- | --- | --- | --- | --- |
| 2022 Mademilov M | Qualitative research | Kyrgyz Republic | 63 Male: 38, Female: 25 | A qualitative study using interviews and focus groups | Mental  Clinical  Social |
| 2020 Huque R | Mixed methods | Bangladesh | 150 (adult in-patients diagnosed with MDR-TB.)  16 (qualitative interviews) | Cross sectional survey using Structured Clinical Interview for Depression (multi-level modelling)  8 patients, 4 carers, 4 health professionals | Mental  Relational social  Clinical  Perception |
| 2019 Laxmeshwar C | Mixed methods | India | 95 | WHOQoL-BREF questionnaire and a separate questionnaire with open-ended questions | Social  Clinical |
| 2017 Khanal S | Qualitative | Nepal | 15 Male :10 , Female: 05 | Semi Structured Interviews (SSIs) | Mental  Psycho-social |
| 2016 Yellappa V | Qualitative | India | 33 | In depth interview | Social – relational  Socioeconomic  Perception |
| 2013 Islam Q.S | Mixed methods | Bangladesh | 1068 | MUAC , BMI | Recovery (Physical)  Malnutrition (Social) |
| 2012 Kaulagekar-Nagarkar A | Qualitative study | India | 113 | Focus Group Discussions | Social Relational  Emotional  Physical |
| 2012 Dr. Emmanuel Atsu Dodor | Qualitative research | Ghana | 40 ( | FGD, SSI | Physical  Psycho- Social |
| 2019 Gyimah FT | Qualitative research | Ghana | 33 | Semi-structured interview guides, | Physical  Mental  Spiritual |

Summary of studies included for Diabetes Mellitus

| Reference (1st author and year) | Design | Location | Participants  Study population  total (M:F) | Tools | Themes looked at |
| --- | --- | --- | --- | --- | --- |
| Korsah KA et al (2022) | Exploratory descriptive qualitative research | Ghana | 27 newly diagnosed Type 2 DM (15:12) | Semi-structured interview | Spiritual |
| Souris KJ (2021) | Qualitative study (done as part of needs assessment done to deliver an intervention) | Bolivia | 18 Type 1 DM (7:11) | Semi-structured interview | Social (relational)  Family support system |
| Alaofe H et al (2021) | Qualitative study (done as part of needs assessment done to deliver an intervention) | Benin | 32 Type 2 DM (20:12) | FGDs | Social (relational) |
| Ansari RM et al (2021) | Qualitative study | Pakistan | 30 Type 2 Diabetes with over 5 years of duration (15:15) | Semi-structured interviews using Chronic Care model as a theoretical framework | Social (Self care & management) |
| Mwila KF et al (2019) | Qualitative descriptive study | Zambia | 28 type 2 DM (11:7) | In-depth interview | Psychosocial  Social (relational)  Social (economical) |
| Amoah VMK et al (2018) | Exploratory qualitative research | Ghana | 107 Type 2 DM (6:4) | Semi-structured interview | Biological  Social (relational)  Spiritual |
| Newlin Lew K (2016) | Qualitative descriptive study | Nicaragua | 42 Type 2 DM | FGDs | Spiritual |
| Belue R (2012) | Qualitative study | Senegal | 54 DM (19:35) | Interviews | Social (relational)  Social (economic) |

Summary of studies included for Schizophrenia

| Reference (1st author and year) | year | Study design | location | Participants Study population   total (M:F) | Gender distribution | Tools | Themes looked at |
| --- | --- | --- | --- | --- | --- | --- | --- |
| Ma M. and Shi Z. and Chen Y. and Ma X | 2023 | Qualitative | China | 11 | 6:5 (F:M) | Indepth Interview | Psycho   Social (relational)  What support system they had |
| Cortez-Vasquez E.V. and Ramos-Calsin L.Y. and Herrera-Lopez V.E.     AO  - Cortez-Vasquez, Esmeralda V. | 2021 | Qualitative | Peru | 52 | 22:30(F:M) | Indepth Interview, FGD | Social (relational) |
| Gamieldien F. and Galvaan R. and Myers B. and Syed Z. and Sorsdahl K. | 2021 | Scoping Review | Turkey, India, China, Swaziland, Indonesia, Egypt, South Africa, Vietnam | 109 full text review | Not mentioned | Literature Review | Bio   Social (relational)   Social (economic)   Spiritual |
| Gandhi S. and Jose D. and Desai G.     AO  - Gandhi, Sailaxmi; | 2020 | Qualitative | India | 18 | 11:7(F:M) | Semi Structured Interview | Bio  Psycho   Social (relational)   Social (economic)   Spiritual |
| Asher L. and Hanlon C. and Birhane R. and Habtamu A. and Eaton J. and Weiss H.A. and Patel V. and Fekadu A. and De Silva M. | 2018 | Mixed method | Ethiopia | 10 | 5:05 | Indepth Interview | Bio Psycho Social (relational) Social (economic) |
| Karambelkar S.S. and Panchal B.N. and Vala A.U. and Kantak A.S. | 2015 | Qualitative | India | 50 | Not mention | Semi Structured Interview | Bio   Social (economic)   Spiritual |
| Sariah A.E. and Outwater A.H. and Malima K.I.Y. | 2014 | Qualitative | Tanzania | 14 | 7:07 | Indepth Interview | Mental Health – (Social relational) |
| Isik I and Ergun G | 2020 | Qualitative | Turkey | 14 | 4:10 (F:M) | Interview – Straus and Corbins approach | Psycho   Social (relational)  What support system they had |
| Koschorke M and Padmavati R and Kumar S and Cohen A and Weiss HA and Chatterjee S and Pereira J and Naik S and John S and Dabholkar H and Balaji M and Chavan A and Varghese M and Thara R and Thornicroft G and Patel V | 2014 | Mixed method | Tamil Nadu, Goa, Maharashtra | 282 persons with schizophrenia, 282 caregivers | 132:150(F:M) | Indepth Interview | Psycho Social (relational) Social (economic) |
| Guner P | 2014 | Qualitative | Istanbul | 9 | 1:08 | Indepth Interview | Bio Psycho Social (relational) Social (economic) Spiritual |
| Can Oz Y and, Unsal Barlas G and Yildiz M | 2019 | Qualitative | Turkey | 33 | Not mentioned | Interview | Socio economic |
| Mall S and Hailemariam M and Selamu M and Fekadu A and Lund C and Patel V and Petersen I and Hanlon C | 2017 | Qualitative | Ethiopia | 70 | 30:40 (F:M) | Indepth Interview and FGDs | Social (relational) Social (economic) |

Table 5. Summary of studies included for HIV

| Reference (1st author and year) | Design | Location | Participants Study population total (M:F) | Tools | Themes looked at |
| --- | --- | --- | --- | --- | --- |
| Islam M.S, 2019 | Qualitative study/ grounded theory | Bangladesh | 19 (8:11) HIV-positive parents who lived with their children, recruited with the support of self-help groups of HIV-positive people | In-depth interviews | -socioeconomic factors  -perception of healing and suffering |
| Hussen S.A., 2014 | multi-method qualitative study | Ethiopia | 20 adults (1:19) | in-depth interviews/Photovoice sessions/ group discussions/ direct participant observations | -perception of healing and suffering  -socioeconomic factors  -mental health |
| Abboah-Offei M / 2020 | Qualitative study | Ghana | 24 (12:12) persons living with HIV/AIDS (PLWHA)   15 health care providers | Semi-structured in-depth interviews | -Clinical measures  -Perceptions of healing  -socioeconomic status  -Quality of life |
| Pierre S / 2017 | Qualitative study | Haiti | 25 (9:16) participants who initiated ART from 2003 through Apr 2004 | Semi-structured in-depth interviews using Ground Theory | -Clinical measures  -Perception of healing and suffering  -Socioeconomic status |
| Kumar S., 2015 | Qualitative study | India | 17 (8:9) adults with HIV affiliated with  formal support networks | 17 in-depth interviews and 4 focus group discussions | -socioeconomic factors |
| E.I. Sianturi, 2020 | Exploratory qualitative study | Indonesia | 13 (5:8) adults living with HIV on ART for at least one year | Semi structured interviews | -socioeconomic factors  -clinical measures |
| Ameli V, 2021 | Social-constructivist qualitative study | Iran | 70 (39:31) adults living with HIV on ART | 12 Individual interviews and 8 focus groups | -Clinical measures  -Socioeconomic status  -Perception of healing and suffering |
| Burns R / 2022 | Qualitative study | Kenya and the Democratic Republic of the Congo | 30 (14:16) hospitalized patients with advanced HIV who had previously initiated first-line ART | In-depth interviews | -Clinical measures  -Mental health  -Perception of healing and suffering |
| Graham SM, 2018 | Qualitative study | Kenya | 30 men living with HIV, on ART for 1 year or longer | In-depth interviews | -clinical measures |
| Tsai AC / 2017 | Longitudinal qualitative study | Kenya | 54 (28:26) persons with HIV (45 in treatment arm and 9 in control arm) | In-depth interviews with 12-month follow-up | -Socioeconomic status  -Mental health |
| Cummings B, 2014 | cross-sectional qualitative evaluation | Mozambique | 70 adults (75% of women) receiving on-going HIV care | semi-structured interviews/ 70 in-depth individual interviews | -socioeconomic factors  -perception of healing and suffering |
| Odediran O.O., 2022 | exploratory qualitative study | Nigeria | 126 women HIV positive on care for at least 12 months | Semi - structured interview, Andersen and Newman’s Behavioral Model for healthcare utilization | -perception of healing and suffering  -socioeconomic factors |
| Rathore M., 2022 | Qualitative study/grounded theory | Pakistan | 12 (10: 1; 1 trans) adults with HIV. 10 were on ART and 2 opted for alternative form of healthcare | Purposive sampling technique, semi-structured interviews | -clinical measures  -mental health  -socioeconomic status |
| Shabalala FS, 2018 | Qualitative study | Swaziland | 52 adults living with HIV who stopped their ART more than 3 months ago | Semi-direct interviews | -clinical measures  -quality of life  -socioeconomic status |
| Shabalala FS, 2018 | Qualitative study | Swaziland | 52 adults living with HIV who stopped their ART more than 3 months ago | Semi-direct interviews | -clinical measures  -quality of life  -socioeconomic status |
| Mandawa M.B, 2022 | Qualitative study/ phenomenological approach | Tanzania | 16 men with HIV on treatment for at least one year | Semi-structured interviews, non-probability, purposive sampling technique | -socioeconomic factors  -perception of healing and suffering |
| Sanga E.S., 2019 | Descriptive qualitative study | Tanzania | 98 (38:60) adults on HIV care between 0 and 6 months | 8 focus group discussions and 10 in-depth interviews | -clinical measures  -socioeconomic factors |
| Ashaba S., 2017 | Qualitative study | Uganda | 20 women (pregnancy and postpartum) | Stratified random sampling  semi-structured interview | -mental health  -socioeconomic factors  -clinical measures |
| Nalugya R /2018 | Qualitative Study | Uganda | 38 (18:20)  HIV-positive parents who had been on ART for over a year | In-depth interviews | -Clinical measures  -socioeconomic status  -Mental health  -Perception of suffering and healing |
| Jennings Mayo-Wilson L / 2020 | Qualitative assessment | Uganda | 42 (13:29) HIV positive adults receiving ART for 2 or more years, and having demonstrated recent adherence problems | Unstructured interviews | -Clinical measures  - |
| Russel S /2019 | Qualitative study | Uganda | 18 male participants who have been on ART for more than 1 year | Two rounds of guided in-depth interviews | -Quality of Life  -Perception of healing and suffering |
| Reynolds Z / 2022 | Qualitative study | Uganda | 18 (9:9) individuals with HIV and 18 (9:9) individuals without HIV | Semi-structured in-depth interviews | -Perception of healing or suffering  -Quality of life  -socioeconomic status |
| Mutabazi-Mwesigire D / 2014 | Qualitative study | Uganda | 20 (5:15) HIV-positive participants | In-depth interviews | -Quality of life  -Socioeconomic status |
| Russel S / 2016 | Qualitative study | Uganda | 38 (18:20) people living iwth HIV and have been on ART for more than one year | Two rounds of in-depth interviews | -Perception of healing and suffering  -Clinical measures  -Mental health  -Quality of life  -Socioeconomic status |
| Buregyeya E, 2017 | Cross-sectional qualitative study | Uganda | 57 pregnant or breastfeeding women living with HIV, either good or poor ART adherers | In-depth interviews | -clinical measures  -socioeconomic status  -perception of healing and suffering |
| Kellett NC, 2016 | Qualitative study | Uganda | 54 women living with HIV, on ART and members of HIV  peer support programmes | Semi-structured interviews and focus groups | -socioeconomic status  -perception of healing and suffering  -clinical measures |
| Bedingfield N, 2014 | Qualitative study | Uganda | 14 adults living with HIV, on Highly active antiretroviral therapy (HAART) between 6 and 12 months | Semi-structured individual and Focus groups interviews, exploratory study using thematic theme analysis | -clinical measures  -mental health  -perception of healing and suffering  -socioeconomic status |
| Ngo V.K., 2013 | Qualitative study/ case study design | Uganda | 40 adults with HIV on ART for at least 6 months | semi-structured interview guide /topic-by-stage framework | -mental health  -clinical measures  -socioeconomic factors  -perception of healing and suffering |
| Isabirye R., 2023 | Qualitative study | Uganda | 25 adults living with HIV, on at least a 24-month ART, with excellent adherence and training in basic psychosocial support for other patients. | In-depth interviews | -clinical measures  -socioeconomic status  -perception of healing and suffering |
| Nguyen MX / 2019 | Qualitative study | Vietnam | 16 (1:15) HIV-infected person with disability (PWID)  8 methadone maintenance treatment providers  4 health officials | Semi-structured in-depth interviews | -mental health |
| Bhadra NM /2020 | Qualitative Study | Vietnam | 20 women (10 with no depression and 10 with probable depression – PHQ-9 score of 10 or above) | In-depth interviews and PHQ-9 | -Mental health  -socioeconomic status  -perception of suffering and healing |
| Moomba K / 2019 | Explorative qualitative study | Zambia | 42 patients on ART (23:19) | Guided FGDs and a semi-structured in-depth interviews | -Clinical Measures  -Socioeconomic status |
| Nixon SA / 2014 | Qualitative study | Zambia | 21 (9:12) people with disabilities (PWDs) who had become HIV-positive, and 11 (7:4) people working with HIV and/or disability | In-depth semi-structured one-on-one interviews | -Clinical measures  -Socioeconomic status  -Quality of life |
| Hanass-Hancock J / 2020 | Longitudinal qualitative study | Zambia | 35 (17:18) adults living with HIV, on ART for 6 months or longer | Three rounds of semi-structured interviews approximately 6 months apart | -Perception of healing and suffering  -Quality of life  -Clinical measures  -Socioeconomic status |
| Nixon SA / 2018 | Longitudinal qualitative study | Zambia | 35 (17:18) adults living with HIV, on ART for 6 months or longer | Three rounds of semi-structured in-depth interviews | -socioeconomic status  -clinical measures  -Perception of healing and suffering  -Quality of life |
| Skovdal M / 2013 | Qualitative study | Zimbabwe | 90 community group members of 9 difference community groups  71 (23:48) in FGDs and 19 (6:13) in in-depth interviews | FGDs and in-depth interviews | -Clinical measures  -Socioeconomic status |

Summary of studies included for Leprosy

| Reference (1 author & Year) | location | Design | Participants      Study population     total (M:F) | Data collection tools | Themes looked at |
| --- | --- | --- | --- | --- | --- |
| Abedi H / 2013 | Pakistan | Qualitative-Purposive sampling | 10 | Semi structured Interview | Physical and social |
| Cross H / 2014 | England | Qualitative | 22 (men -21, Women - 1) | FGD | Physical and social |
| Lusli M / 2015 | United States | Qualitative | 14 | SSI and FGD guiding questions | Physical and social |
| da Silva Santos / 2015 | Brazil | Qualitative | 8 | FGD, Literature review | Physical, Mental and Social |
| Hofstraat K. / 2015 | Amsterdam 1097 DN, Netherlands.   United Kingdom | Qualitative , Literature Review | Male: 190, Female-185 | Literature Review- PsycINFO and Web of Knowledge | Physical and social |
| Peters R.M.H. / 2016 | United Kingdom | Qualitative (Vidoe graphy) | 12 | Interview | Social |
| Dadun D / 2017 | England | Quantitative : . To achieve adequate power for the quantitative assessment and the   comparisons between the baseline and the end survey we calculated a required sample size of   600 people affected by leprosy (150 per study area) and 200 community members (50 per   study area). | 600 | Questionnaire based interview | Social |
| Azad-uz-zaman Q. / 2017 | Bangladesh | Qualitative and Cross sectional study | 92 (M-56, F-36) | Semi Structured Intereview | Physical and Mental |
| Govindharaj P/2018 | India | Quantitative and Qualitative | 358 (Male - 212 , Female-146) | Questionnaire & Interview | Physical and Mental |
| Pinheiro M.G.C. / 2018 | Brazil | Qualitative - | 13 publications | Bibliographical survey: following databases: SCOPUS, Cumulative Index to Nursing and Allied    Health Literature (CINAHL), and PUBMED, accessed through the Capes Journal Portal, using descriptors indexed    in Medical Subject Headings (MeSH); | Physical |
| Vieira C.S.C.A. / 2018 | India | Qualitative & Quantitative | 210 | The sampling selection was    performed on the database of the Information    System on Diseases of Compulsory Declaration. | Social |
| Dwivedi P /2018 | India | Qualitative | 40 | Observation & Indepth Interview | Social |
| Pinheiro MGC / 2019 | Brazil |  |  |  |  |
| Sottie / 2019 | Ghana | Qualitative | 20 | FGD and Indepth Interview | Mental and Spiritual |
| Da Silva /2019 | Brazil. | Qualitative | 21 | Semi Structured Interview - home visits and phone calls | Physical and Social |
| Van'T Noordende /2019 | Brazil | Literature Review | 28 | Literature Review in 3 phases |  |
| Dadun D. /2019 | Indonesia, | Quantitative & Qualitative :  Randomized-controlled mixed-methods | 369 | Baseline and Endline quantitative and qualitative study.EPI Info Software &  Indepth Interview.   WHOQOF BREIF | Mental and Social |
| Somar P/2020 | England | Qualitative | 65 | PubMed, Web of Science, Scopus, PsycINFO, Infolep and   InfoNTD |  |
| van Dorst/2020 | United States | Quantitative | 196 | Mental wellbeing status (WEMWBS) , level of depression (PHQ-9), level of stigma (5-QSI-AP) | Mental and Social |
| Rai S.S. /2020 | Indonesia | Qualitative | 40 (Male-18, Female-21, Transgender-1) | Questionnaires | Physical, Mental, Social and Spiritual |
