# Supplementary material for: Understanding healing: A comparative analysis in chronic diseases with leprosy—A scoping review
Source: PLoS Negl Trop Dis. 2026 Mar 2;20(3):e0013748. doi: 10.1371/journal.pntd.0013748 (PMC12962515; doi:10.1371/journal.pntd.0013748)
Supplement: S2 Table — This table synthesizes findings from 85 studies, categorized by five key dimensions of healing—physical, psychological, social (relational), socioeconomic, and spiritual—across five chronic conditions: leprosy, tuberculosis, diabetes mellitus, HIV/AIDS, and schizophrenia. (DOCX) [file pntd.0013748.s002.docx]

# S2 Table. Summary of outcomes across chronic diseases included in the scoping review

This table synthesizes findings from 85 studies analyzed in the scoping review.

Evidence was categorized by five key dimensions of healing—physical, psychological, social (relational), socioeconomic, and spiritual—across five chronic conditions: leprosy, tuberculosis, diabetes mellitus, HIV/AIDS, and schizophrenia.

| Disease | Physical / Clinical | Social (Relational) | Socioeconomic | Psychological | Spiritual / Faith-based |
| --- | --- | --- | --- | --- | --- |
| Leprosy | Education and self-awareness improved self-care and reduced disability-related risks. | Social exclusion and stigma led to fear, anxiety, and depression. Counselling and group bonding fostered social participation and empowerment. | Economic development programs (e.g., microcredit) improved happiness and opportunities. Women faced unique workplace stigma. | Shame, low self-esteem, and mental stress affected resilience; peer support and self-care engagement built confidence. | Spirituality and group bonding promoted resilience; family and community acceptance supported spiritual healing. |
| Tuberculosis (TB) | Persistent post-treatment depression and confusion around medication adherence were common. | Stigma from family and community diminished dignity; family and workplace support were crucial to healing. | Poverty and low-caste status intensified financial hardship, leading to debt and income loss. | Psychological distress was pervasive throughout treatment and recovery; positive thinking promoted adherence. | Belief in divine healing provided strength and reduced fear of death. |
| HIV / AIDS | Physical recovery important, though long-term health anxieties persisted. ART adherence improved with community-based delivery. | Family and peer support improved adherence and reduced stigma; community groups gave belonging. | Employment and income stability improved adherence and wellbeing. | Acceptance of HIV status and finding purpose reduced suffering; self-stigma remained a barrier. | Spirituality both hindered and supported healing; faith enhanced motivation and treatment adherence when combined with social support. |
| Diabetes Mellitus | Diet and medication adherence were difficult due to cultural food norms. | Peer support groups and family networks improved coping; stigma limited open discussion. | Family played major financial and practical support roles. | Emotional distress from dietary restrictions and chronic stress common; resilience linked to family and faith. | Prayer and faith perceived as helping control blood sugar and giving hope. Acceptance as ‘God’s plan’ fostered adaptation. |
| Schizophrenia | Medication adherence viewed as essential by some and a sign of progress by others. | Social relationships, family connection, and community support facilitated recovery. | Economic difficulties due to unemployment or underemployment common. | Hope, optimism, and self-awareness were central to recovery; low self-esteem was a barrier. | Spirituality and religion promoted resilience and psychological insight; faith communities provided comfort and purpose. |

Caption: This table corresponds to the narrative synthesis summarized in Table 7 of the main text and supports PRISMA-ScR item 15 by describing outcome themes across evidence sources. Healing dimensions include physical, psychological, social (relational), socioeconomic, and spiritual domains.
