# Supplementary material for: Understanding healing: A comparative analysis in chronic diseases with leprosy—A scoping review
Source: PLoS Negl Trop Dis. 2026 Mar 2;20(3):e0013748. doi: 10.1371/journal.pntd.0013748 (PMC12962515; doi:10.1371/journal.pntd.0013748)
Supplement: S3 Table — This table provides a detailed mapping of the 22-item PRISMA-ScR checklist to the corresponding sections of the manuscript. (DOCX) [file pntd.0013748.s003.docx]

# S3 Table. PRISMA-ScR 2020 Checklist

This table provides a detailed mapping of the 22-item PRISMA-ScR checklist to the corresponding sections of the manuscript titled Understanding Healing: A comparative analysis in Chronic Diseases with leprosy — A Scoping Review

| Item No | PRISMA-ScR Checklist Item | Reported (Yes/No) | Location in Manuscript (Section) |
| --- | --- | --- | --- |
| 1 | Title: Identify the report as a scoping review. | Yes | Title |
| 2 | Abstract: Provide a structured summary including background, objectives, eligibility criteria, sources of evidence, charting methods, results, and conclusions. | Yes | Abstract |
| 3 | Rationale: Describe the rationale for the review in the context of existing knowledge. | Yes | Introduction |
| 4 | Objectives: Provide explicit statement of questions and objectives. | Yes | Introduction (Aim and objectives) |
| 5 | Protocol and registration: Indicate if a review protocol exists and where it can be accessed. | No | Methods |
| 6 | Eligibility criteria: Specify characteristics of sources of evidence and rationale. | Yes | Methods (Eligibility Criteria) |
| 7 | Information sources: Describe all information sources in the search and date last searched. | Yes | Methods (Information sources and Search Strategy) |
| 8 | Search: Present full search strategy for at least one database. | Yes | Supplementary Table 1 |
| 9 | Selection of sources: State the process for selecting sources of evidence (screening and eligibility). | Yes | Methods (Selection of Sources) |
| 10 | Data charting process: Describe methods of charting data from included sources. | Yes | Methods (Data Charting Process) |
| 11 | Data items: List and define all variables for which data were sought. | Yes | Methods (Data Charting Process) |
| 12 | Critical appraisal of individual sources of evidence (if done): Report methods and rationale. | No (Not applicable) | Methods (Synthesis of Results) |
| 13 | Synthesis of results: Describe methods of handling and summarizing the data. | Yes | Methods (Synthesis of Results) |
| 14 | Selection of sources of evidence: Give numbers of sources screened, assessed for eligibility, and included, with reasons for exclusions at each stage, ideally using a flow diagram. | Yes | Results (PRISMA-ScR flow diagram, Figure 1) |
| 15 | Characteristics of sources of evidence: Present characteristics of included sources. | Yes | Results (S1 Table) |
| 16 | Critical appraisal within sources of evidence: If done, present data. | No | Methods / Results |
| 17 | Results of individual sources of evidence: For each included source, present the relevant data. | Yes | Results and S1 Table |
| 18 | Synthesis of results: Summarize the main results by theme, and consider relevance to objectives. | Yes | Results and Discussion (5D Framework) |
| 19 | Limitations: Discuss limitations of the scoping review process. | Yes | Limitations section |
| 20 | Conclusions: Provide general interpretation of the results with implications for practice/policy/research. | Yes | Conclusion |
| 21 | Funding: Describe sources of funding and role of funders. | No | End of manuscript |
| 22 | Competing interests: Declare any competing interests of authors. | No | End of manuscript |
