# Supplementary figures and images for: Understanding healing: A comparative analysis in chronic diseases with leprosy—A scoping review
Source: PLoS Negl Trop Dis. 2026 Mar 2;20(3):e0013748. doi: 10.1371/journal.pntd.0013748 (PMC12962515; doi:10.1371/journal.pntd.0013748)

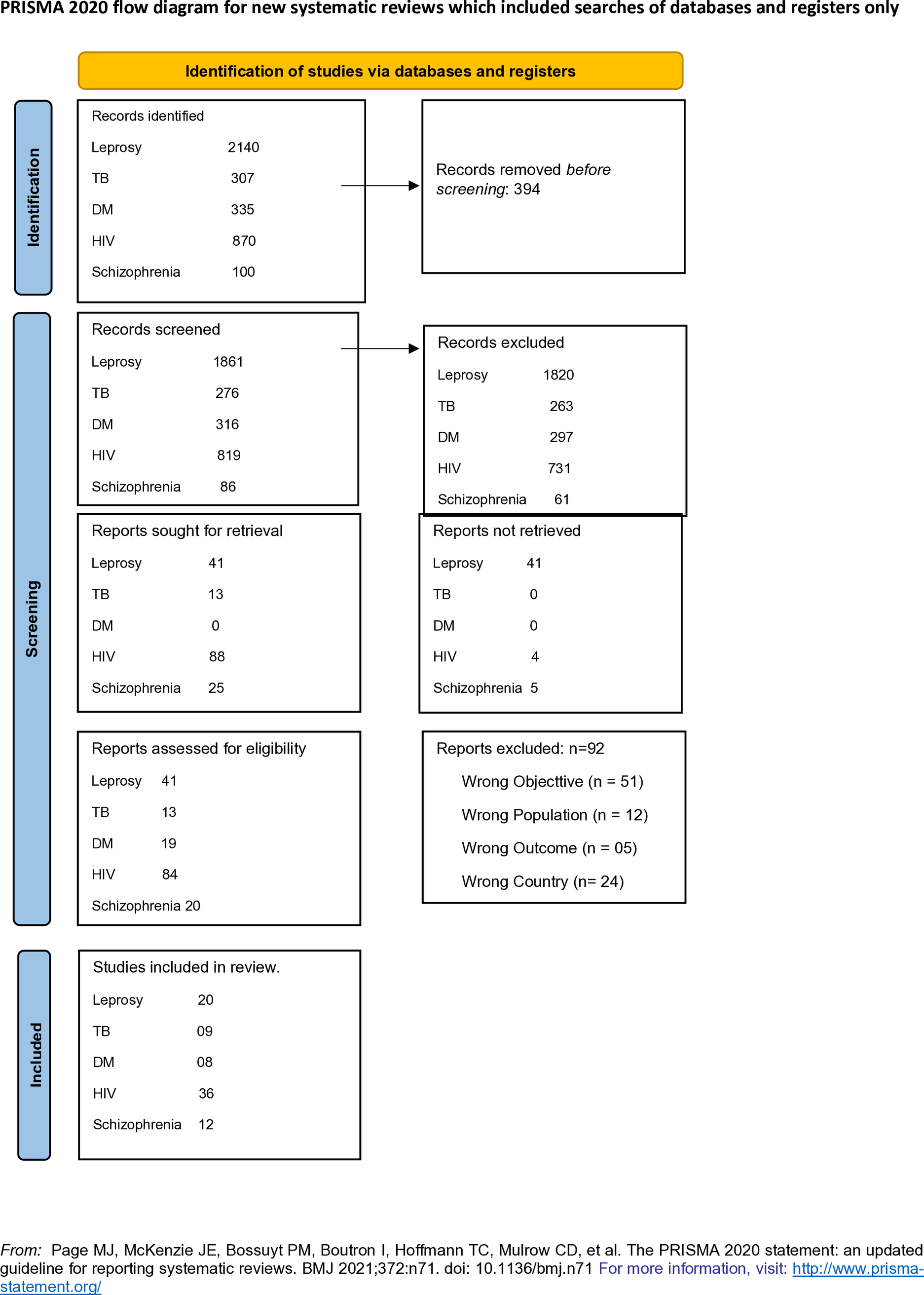

Supplement: S1 Prisma Diagram — (TIF) [file pntd.0013748.s004.tif]
