# Supplementary material for: Understanding healing: A comparative analysis in chronic diseases with leprosy—A scoping review
Source: PLoS Negl Trop Dis. 2026 Mar 2;20(3):e0013748. doi: 10.1371/journal.pntd.0013748 (PMC12962515; doi:10.1371/journal.pntd.0013748)
Supplement: Attachment — Submitted filename: Response letter.docx [file pntd.0013748.s006.docx]

Dear Editorial Team,

Thank you for reviewing our submission, *“Understanding Healing: A Comparative Analysis in Chronic Diseases with Leprosy — A Scoping Review.”*

We thank you for allowing us to address the requested checks. Please find our responses below:

1. The CRediT author contributions for all authors have been completed accurately and in full.
2. The duplicated and unnecessary cover letter files have been removed. Only files relevant to the current version of the manuscript are now included.
3. We have reviewed the amended Competing Interest statement and confirm that it is correct.
4. The author list in the manuscript file now matches exactly with the author list in the online submission form, including order and spelling of names.
5. All figure files have been labelled and uploaded in numerical order.
6. Figure 2 has been revised and is correctly uploaded. The figure is appropriately referenced in the text.
7. Figure 0 has been deleted from the file inventory. All remaining figures now have corresponding in-text citations and legends in the manuscript.
8. All tables have been removed as individual files and are now included within the manuscript as editable, cell-based tables, in accordance with journal guidelines.
9. The PRISMA-ScR Flow Diagram has now been uploaded as Supporting Information with the appropriate item designation.
10. A complete list of legends for all Supporting Information files has been added after the references section, including the PRISMA-ScR Checklist (S3 Table).
11. The Financial Disclosure statement has been revised and completed in full sentences using the exact wording intended for publication.

We hope these revisions fully address the editorial requirements. Please let us know if any further clarification or modification is needed.

Thank you for your support.

Sincerely,
Dr. Joydeepa Darlong
On behalf of all authors

29/01/2026
